# Serum 25-Hydroxyvitamin D Levels in Breast Cancer Patients: A Cross-Sectional Analysis by Molecular Tumor Subtypes

**DOI:** 10.3390/jcm15020833

**Published:** 2026-01-20

**Authors:** Dorota Weber, Andrzej Stanisławek, Anna Irzmańska-Hudziak, Teresa Kulik, Anna Beata Pacian, Monika Baryła-Matejczuk, Marta Łuczyk, Robert Łuczyk

**Affiliations:** 1 Institute of Human Sciences, WSEI University, 20-209 Lublin, Polandmonika.baryla@wsei.pl (M.B.-M.); 2 St. John of Dukla Centre of Oncology of Lublin Region, 20-090 Lublin, Poland; 3 Institute of Health Sciences, Catholic University of Lublin, 20-950 Lublin, Poland; 4 Department of Health Education, Faculty of Nursing Development, Medical University in Lublin, 20-059 Lublin, Poland; 5 Department of Long-Term Care Nursing, Faculty of Health Sciences, Medical University of Lublin, 20-093 Lublin, Poland; 6 Department of Internal Medicine and Internal Nursing, Faculty of Health Sciences, Medical University of Lublin, 20-059 Lublin, Poland

**Keywords:** vitamin D, breast cancer, molecular subtypes, HER2, seasonal variation

## Abstract

**Background**: Vitamin D deficiency has been implicated in breast cancer pathogenesis and prognosis. However, the relationship between serum 25-hydroxyvitamin D [25(OH)D] levels and molecular breast cancer subtypes remains incompletely understood. **Methods**: This cross-sectional study included 168 women (89 breast cancer patients, 79 healthy controls) from Poland. Serum 25(OH)D was measured by electrochemiluminescence immunoassay. Blood samples were collected year-round, with 54% obtained during winter/spring months (October–March). Molecular subtypes (luminal A, luminal B, HER2-enriched, triple-negative) were classified by immunohistochemistry. **Results**: Mean 25(OH)D was 30 ± 13 ng/mL, with 55% showing insufficiency (<30 ng/mL). No significant differences were observed between patients and controls (*p* = 0.93). A borderline non-significant trend was observed across molecular subtypes (*p* = 0.055). HER2-enriched tumors showed descriptively higher concentrations (37.6 ng/mL, 95% CI: 29.5–45.8) compared to luminal A (31.0 ng/mL), luminal B (26.4 ng/mL), and triple-negative (25.9 ng/mL). A significant subtype × season interaction was detected (*p* = 0.015), though interpretation is limited by the absence of a main seasonal effect (*p* = 0.64). Age (OR = 1.06, *p* = 0.023) and BMI (OR = 1.06, *p* = 0.090) predicted vitamin D deficiency. **Conclusions**: Vitamin D insufficiency is prevalent in breast cancer patients and healthy women. In this exploratory analysis with limited statistical power, no definitive associations between 25(OH)D and molecular subtype were established. The descriptive trend suggesting higher vitamin D in HER2-enriched tumors requires validation. Limitations: Small sample sizes (*n* = 11–35 per subtype) and post-surgical blood collection limit interpretation; findings require validation in larger cohorts.

## 1. Introduction

Breast cancer represents a leading cause of cancer-related morbidity and mortality worldwide, with significant variations in incidence, treatment response, and prognosis across different molecular subtypes [1]. In Poland, breast cancer accounts for 22.5% of all female malignancies, with age-adjusted incidence rates continuing to increase over the past three decades [2]. The heterogeneous nature of breast cancer, characterized by distinct molecular profiles including luminal A, luminal B, HER2-enriched, and triple-negative subtypes, necessitates personalized approaches to prevention, treatment, and supportive care [3].

Vitamin D, primarily recognized for its role in calcium homeostasis and bone metabolism, has emerged as a molecule of interest in cancer research due to its pleiotropic biological functions [4]. The active metabolite, 1,25-dihydroxyvitamin D3, exerts anticancer effects through multiple mechanisms, including regulation of cell proliferation, differentiation, apoptosis, and immune modulation [5]. Serum 25-hydroxyvitamin D [25(OH)D], the major circulating metabolite and established biomarker of vitamin D status, reflects both dietary intake and cutaneous synthesis [6].

Epidemiological studies have demonstrated inverse relationships between circulating 25(OH)D levels and breast cancer incidence across multiple populations. Prospective analyses reveal dose–response relationships, with meta-analyses indicating that each 10 ng/mL increment in serum 25(OH)D is associated with an approximately 4–9% reduction in breast cancer risk [7,8]. A pooled analysis of cohort studies demonstrated that women with 25(OH)D concentrations ≥ 60 ng/mL had a substantially lower breast cancer risk compared to those with concentrations < 20 ng/mL. However, the relationship appears complex and potentially modified by factors including menopausal status, geographic latitude, body composition, and tumor characteristics [9].

Beyond incidence, vitamin D status has been associated with survival outcomes. Meta-analyses demonstrate that vitamin D sufficiency is associated with improved overall survival and disease-free survival in breast cancer patients [9,10], suggesting potential clinical relevance of vitamin D status throughout the cancer care trajectory.

The Polish population demonstrates particularly high rates of vitamin D insufficiency, with epidemiological surveys indicating that 89.9% of adults maintain serum 25(OH)D concentrations below 30 ng/mL [11]. This widespread insufficiency, combined with increasing breast cancer incidence, underscores the clinical relevance of investigating vitamin D status in Polish breast cancer patients. Factors known to influence vitamin D status include age, body mass index, season of blood collection, and sun exposure behaviors [12].

Molecular classification of breast cancer based on hormone receptor expression and HER2 status has revolutionized treatment approaches and prognostic stratification [13]. Luminal A tumors (estrogen receptor-positive, progesterone receptor-positive, HER2-negative, low Ki-67) generally demonstrate a favorable prognosis, while triple-negative (estrogen receptor-negative, progesterone receptor-negative, and HER2-negative) and HER2-enriched subtypes are associated with more aggressive clinical behavior [14]. Emerging mechanistic evidence suggests that vitamin D receptor (VDR) expression may vary across molecular subtypes, potentially influencing hormone receptor status and HER2 expression through complex signaling pathways [15]. Some clinical studies have reported differential outcomes with vitamin D supplementation across subtypes, with HER2-positive patients showing improved outcomes during adjuvant chemotherapy when receiving supplementation [16], while vitamin D deficiency has been associated with poor outcomes, specifically in luminal-type breast cancer in some cohorts [17]. However, whether vitamin D status at diagnosis differs across molecular subtypes remains inconsistent across studies, potentially reflecting differences in study design, timing of blood collection, population characteristics, and sample sizes.

This study aimed to evaluate serum 25(OH)D concentrations in breast cancer patients compared to healthy controls, with particular emphasis on potential associations with molecular tumor subtypes in a Central European population. We examined factors known to influence vitamin D status, including age, menopausal status, body mass index (BMI), and season of blood collection. Given the post-surgical timing of blood collection in our study, findings should be interpreted as exploratory observations that may inform future research design rather than definitive assessments of pre-diagnostic vitamin D status and cancer risk.

## 2. Materials and Methods

### 2.1. Study Design and Participants

This cross-sectional study was conducted at the St. John of Dukla Centre of Oncology of the Lublin Region (COZL) between 2018 and 2019. The study protocol was approved by the institutional ethics committee, and all participants provided written informed consent in accordance with the Declaration of Helsinki.

Inclusion criteria for breast cancer patients included (1) histologically confirmed invasive breast carcinoma, (2) completion of surgical treatment, (3) availability of molecular subtype classification based on immunohistochemistry, and (4) age ≥ 18 years. Exclusion criteria included (1) metastatic disease, (2) concurrent malignancy, (3) self-reported vitamin D supplementation within 3 months prior to enrollment, and (4) severe renal or hepatic dysfunction that could affect vitamin D metabolism.

Control participants were recruited from healthy women attending outpatient clinics at the same institution. Control inclusion criteria included (1) no history of malignancy, (2) normal clinical breast examination, and (3) age-matched to the patient population. The same exclusion criteria applied to control subjects.

### 2.2. Data Collection

Demographic and clinical data were collected through standardized questionnaires and medical record review. Variables included age, menopausal status, height, weight, and season of blood collection. Body mass index (BMI) was calculated as weight (kg) divided by height squared (m^2^) and categorized according to WHO criteria: underweight (<18.5), normal weight (18.5–24.9), overweight (25.0–29.9), and obese (≥30.0 kg/m^2^). Menopausal status was defined as cessation of menstruation for ≥12 consecutive months or prior bilateral oophorectomy.

### 2.3. Blood Sample Collection and Timing

Blood samples were obtained from breast cancer patients following completion of surgical treatment. The timing of blood collection ranged from 2 to 8 weeks post-surgery, with samples collected during routine post-operative follow-up visits. For control participants, blood samples were collected during routine outpatient clinic visits. All blood samples were drawn in the morning (between 8:00 a.m. and 11:00 a.m.) after an overnight fast to minimize diurnal variation, processed within 2 h of collection, and stored until analysis.

### 2.4. Laboratory Analysis

Serum 25-hydroxyvitamin D [25(OH)D] concentrations were measured using electrochemiluminescence immunoassays (Vitamin D Total Test, Roche Diagnostics, Mannheim, Germany) on the Cobas e410 analyzer (Roche Diagnostics, Mannheim, Germany).. This assay measures both 25(OH)D2 and 25(OH)D3 with an analytical sensitivity of 3.0 ng/mL and functional sensitivity (20% CV) of 7.5 ng/mL. Intra-assay and inter-assay coefficients of variation are <6.5% and <10%, respectively. The assay is standardized against the National Institute of Standards and Technology (NIST) reference materials.

Vitamin D status was categorized according to widely accepted clinical thresholds: deficiency (<20 ng/mL), insufficiency (20–29 ng/mL), and sufficiency (≥30 ng/mL). For some analyses, a dichotomous classification was used with insufficiency defined as serum 25(OH)D < 30 ng/mL [18].

### 2.5. Molecular Subtype Classification

Molecular subtypes were determined through immunohistochemistry (IHC) for estrogen receptor (ER), progesterone receptor (PR), human epidermal growth factor receptor 2 (HER2), and Ki-67 proliferation index, performed on formalin-fixed paraffin-embedded tumor tissue. HER2 status was supplemented by fluorescence in situ hybridization (FISH) when IHC results were equivocal (2+). Hormone receptor positivity was defined as ≥1% nuclear staining according to current ASCO/CAP guidelines.

Breast cancer molecular subtypes were classified as follows:

- Luminal A: ER+ and/or PR+ (≥1%), HER2−, Ki-67 < 20%;
- Luminal B: ER+ and/or PR+ (≥1%), either HER2+ or HER2− with Ki-67 ≥ 20%;
- HER2-enriched: ER−, PR− (<1%), HER2+;
- Triple-negative: ER−, PR−, HER2−.

### 2.6. Statistical Analysis

Descriptive statistics were calculated as means ± standard deviations for continuous variables and frequencies with percentages for categorical variables. The breast cancer and control groups were compared in terms of their characteristics using Student’s *t*-test and Pearson’s chi-square test. Given that each group included more than 30 participants, a normal distribution was assumed, and Levene’s test indicated no violation of the homogeneity of variance assumption (*p* > 0.05).

Vitamin D levels were analyzed using a univariate general linear model (GLM) framework. Molecular subtype of breast cancer and season (summer vs. winter) were included as fixed factors, with season serving to adjust the main effects for potential confounding. An interaction term between molecular subtype and season was included to test whether the effect of season varied across subtypes. Post hoc comparisons were performed with the Unequal N HSD test where appropriate. The Shapiro–Wilk test indicated that the distribution of vitamin D levels did not substantially deviate from normality within the molecular subtype groups. For subtypes with smaller sample sizes (*n* < 30), the Shapiro–Wilk results were used directly to assess normality, whereas for larger subgroups (*n* ≥ 30), approximate normality was assumed based on the central limit theorem. Levene’s test confirmed that the assumption of homogeneity of variances was met (*p* > 0.05).

Multivariable logistic regression analysis was performed to identify independent predictors of vitamin D deficiency, with odds ratios (ORs) and 95% confidence intervals (CIs) calculated to enhance clinical interpretation of the original findings.

Statistical significance was set at *p* < 0.05. All analyses were conducted using SPSS version 26.0 (IBM Corporation, Armonk, NY, USA).

### 2.7. Use of Generative Artificial Intelligence

Generative artificial intelligence (Claude 3.5 Sonnet, Anthropic, San Francisco, CA, USA) was used to assist with literature review, statistical interpretation guidance, and language refinement. All AI-generated content was carefully reviewed, verified against primary sources, and edited by the authors to ensure accuracy and appropriateness. AI was not used for data collection, analysis, or interpretation of results. The final intellectual content and scientific conclusions are solely the responsibility of the authors.

## 3. Results

### 3.1. Participant Characteristics

The final analytical cohort comprised 168 women: 89 breast cancer patients (53%) and 79 healthy controls (47%). Mean age was 54 ± 10 years, with no significant difference between groups (*p* = 0.094). The majority of participants were postmenopausal (66%), with similar distributions across groups (*p* = 0.12).

Blood samples were collected year-round. Winter and spring months (October through March) accounted for 54% of sampling (*n* = 90), when vitamin D synthesis from UVB exposure is substantially reduced at Central European latitudes. Summer and fall months (April through September) accounted for 46% (*n* = 78). The distribution of seasonal sampling did not differ significantly between breast cancer patients and controls (*p* = 0.10).

Mean BMI was 27 ± 5 kg/m^2^, with 43% of participants demonstrating normal weight (BMI 18.5–24.9), 38% overweight (BMI 25.0–29.9), and 19% obesity (BMI ≥ 30.0). BMI did not differ significantly between breast cancer patients and controls. Among breast cancer patients, molecular subtype distribution was: luminal A (39%, *n* = 35), luminal B (34%, *n* = 30), triple-negative (15%, *n* = 13), and HER2-enriched (12%, *n* = 11). Demographic and clinical characteristics stratified by group are presented in Table 1.

### 3.2. Vitamin D Status in Breast Cancer Patients vs. Controls

Overall mean serum 25(OH)D concentration was 30 ± 13 ng/mL (range: 7.0–74.0 ng/mL). Using the threshold of <30 ng/mL to define vitamin D insufficiency, 55% of participants (*n* = 93) demonstrated suboptimal status. When stratified by conventional clinical categories, 32% had deficiency (<20 ng/mL), 23% had insufficiency (20–29 ng/mL), and 45% had sufficiency (≥30 ng/mL). No statistically significant difference in mean 25(OH)D concentrations was detected between breast cancer patients and healthy controls (30 ± 14 vs. 29 ± 11 ng/mL, *p* = 0.93) (Table 2). Similarly, the prevalence of vitamin D insufficiency (<30 ng/mL) did not differ significantly between groups (55% in breast cancer patients vs. 56% in controls).

### 3.3. Molecular Subtype Analysis

Among breast cancer patients, vitamin D status varied across molecular subtypes, with the lowest levels observed in the luminal B (M = 26.40, SE = 2.47) and triple-negative (M = 25.92, SE = 3.75) subtypes, and the highest in the HER2-enriched subtype (M = 37.64, SE = 4.08). However, these differences were not statistically significant (*p* = 0.091) (Table 3).

### 3.4. Seasonal Variation and Subtype Interactions

**Figure 1:**
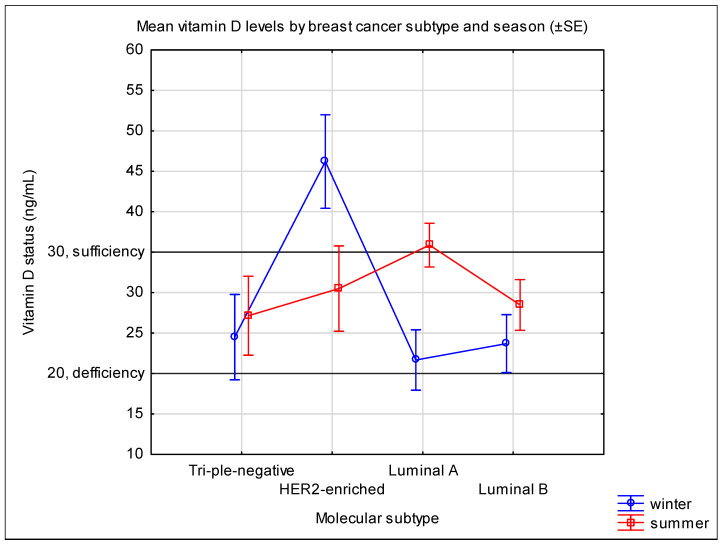
Mean vitamin D levels by breast cancer subtype and season (±SE).

Univariate ANOVA showed a trend toward a main effect of molecular subtype on vitamin D levels (F(3,81) = 2.64, *p* = 0.055), adjusted for season. The main effect of season was not significant (F(1,81) = 0.22, *p* = 0.64). Importantly, a significant interaction between molecular subtype and season was observed (F(3,81) = 3.7, *p* = 0.015), indicating that the influence of season on vitamin D levels differed depending on the molecular subtype. Post-hoc comparisons (Unequal N HSD) did not reveal statistically significant pairwise differences between specific subtype–season combinations (all *p* > 0.05), although a trend was observed between non-luminal and luminal A subtypes in winter (*p* = 0.067) (Table 4, Figure 1).

Mean 25-hydroxyvitamin D [25(OH)D] concentrations across breast cancer molecular subtypes stratified by season of blood collection. Error bars represent standard error (SE). A statistically significant interaction between molecular subtype and season was observed (F(3,81) = 3.70, *p* = 0.015), though this finding should be interpreted cautiously given the absence of a significant main effect of season (*p* = 0.64) and very small sample sizes in some subtype–season combinations (*n* = 3–18). Post-hoc pairwise comparisons did not reveal statistically significant differences (all *p* > 0.05), though a trend was observed between HER2-enriched and luminal A subtypes in winter (*p* = 0.067). TN, triple-negative; HER2, HER2-enriched; LA, luminal A; LB, luminal B.

### 3.5. Predictors of Vitamin D Insufficiency

Logistic regression analysis indicated that age was a significant predictor of vitamin D deficiency, with each additional year of age increasing the odds of deficiency by approximately 6% (OR = 1.06, 95% CI: 1.01–1.12, *p* = 0.023). Although BMI, winter season, menopausal status, and group were not statistically significant predictors, some trends were observed: higher BMI was associated with a modest increase in the odds of deficiency (OR = 1.06, 95% CI: 0.99–1.14, *p* = 0.09), postmenopausal women tended to have lower odds of deficiency compared with premenopausal women (OR = 0.45, 95% CI: 0.16–1.31, *p* = 0.15), and patients assessed in winter had slightly higher odds of deficiency than those assessed in summer (OR = 1.20, 95% CI: 0.64–2.26, *p* = 0.58). No trend was observed for group membership (OR = 1.03, 95% CI: 0.55–1.96, *p* = 0.92) (Table 5).

## 4. Discussion

### 4.1. Principal Findings

This cross-sectional exploratory study of Polish women demonstrated widespread vitamin D insufficiency affecting 55% of participants, with no significant differences in serum 25(OH)D concentrations between breast cancer patients and healthy controls (29.5 vs. 29.4 ng/mL, *p* = 0.93). The mean 25(OH)D concentration of 30 ng/mL closely approximates the threshold distinguishing insufficiency from sufficiency (30 ng/mL), indicating marginal vitamin D status across the study population. In exploratory analyses examining molecular breast cancer subtypes, a borderline non-significant trend (*p* = 0.055) suggested potentially higher 25(OH)D concentrations in HER2-enriched tumors (37.6 ng/mL) compared to other subtypes (26–31 ng/mL), though small sample sizes (*n* = 11–35 per subtype) and wide confidence intervals preclude definitive interpretation.

### 4.2. Interpretation of Vitamin D Status Findings in Breast Cancer Versus Control

The absence of significant differences in 25(OH)D levels between breast cancer patients and controls contradicts several previous studies suggesting lower vitamin D concentrations in cancer populations [19,20]. However, our findings align with other investigations that failed to demonstrate significant associations between vitamin D status and breast cancer diagnosis [21]. The discrepancy may reflect several factors, including variations in study design (prospective vs. cross-sectional), population characteristics (baseline vitamin D status, dietary patterns, and supplementation practices), timing of blood collection relative to diagnosis and treatment, and unmeasured confounding variables.

Post-surgical inflammatory responses may acutely affect vitamin D metabolism and circulating concentrations. More fundamentally, our measurements may not reflect pre-diagnostic status if vitamin D plays a causal role in breast cancer development. Post-diagnosis behavioral changes could have altered vitamin D concentrations. These include reduced physical activity and sun exposure, dietary modifications, or initiation of supplementation. This temporal disconnect between exposure measurement and the relevant etiologic window substantially limits causal inference and may explain the null findings. Future investigations should prioritize pre-diagnostic sampling from prospective cohorts or, at a minimum, sample collection at diagnosis prior to surgical or systemic treatment. Logistic regression analysis confirmed that breast cancer patients did not differ from controls in odds of vitamin D insufficiency after adjusting for age, BMI, season, and menopausal status (OR = 1.03, 95% CI: 0.55–1.96, *p* = 0.92), reinforcing the null association observed in univariate comparisons.

### 4.3. Vitamin D Status and Breast Cancer Molecular Subtypes

The relationship between vitamin D status and breast cancer molecular subtypes remains an area of active investigation with conflicting findings across studies. Our exploratory analysis revealed a borderline non-significant trend (*p* = 0.055) suggesting possible differences across subtypes, with HER2-enriched tumors showing descriptively higher mean 25(OH)D concentrations (37.6 ng/mL, 95% CI: 29.5–45.8) compared to luminal A (31.0 ng/mL), luminal B (26.4 ng/mL), and triple-negative subtypes (25.9 ng/mL). This trend did not reach conventional statistical significance and should be interpreted with extreme caution. Small sample sizes (*n* = 11 for HER2-enriched, *n* = 13 for triple-negative) and extremely wide confidence intervals (HER2-enriched 95% CI spans 16.3 ng/mL) limit interpretation. However, if validated in adequately powered cohorts, the observed pattern could align with emerging mechanistic evidence. Zhang et al. (2017) demonstrated that vitamin D receptor (VDR) expression varies across molecular subtypes and influences hormone receptor status and HER2 expression through complex genomic pathways [15]. Some clinical studies have reported improved outcomes with vitamin D supplementation, specifically in HER2-positive breast cancer patients undergoing adjuvant chemotherapy (Zeichner et al., 2015) [16], suggesting potential subtype-specific biological interactions. The elevated 25(OH)D levels we observed in HER2-enriched tumors, though preliminary and requiring validation, merit further investigation in this mechanistic context.

However, substantial uncertainty surrounds these findings. The borderline *p*-value (0.055), while approaching the conventional α = 0.05 threshold, does not meet the criterion for statistical significance. More importantly, the small sample sizes resulted in extremely wide confidence intervals that encompass both null effects and potentially clinically meaningful differences. The borderline *p*-value most likely reflects inadequate statistical power rather than a genuine biological difference. Previous literature on vitamin D status across breast cancer molecular subtypes has yielded inconsistent results. Kim et al. (2011) reported that vitamin D deficiency correlated with poor outcomes specifically in patients with luminal-type breast cancer [17], while other investigations have found no associations between vitamin D status and molecular subtype classification. These discrepancies likely reflect differences in study design, population characteristics, sample size adequacy, timing of blood collection, and control for confounding factors, including supplementation and seasonal variation.

When vitamin D status was categorized dichotomously (insufficiency < 30 ng/mL vs. sufficiency ≥ 30 ng/mL), prevalence of insufficiency ranged from 45% in HER2-enriched tumors to 63% in luminal B tumors, though these proportional differences were not statistically significant (chi-square *p* = 0.70). While the lowest insufficiency rate in HER2-enriched tumors is consistent with this subtype’s descriptively higher mean 25(OH)D concentration, the wide range of prevalence estimates (45–63%) across relatively small groups suggests substantial sampling variability.

### 4.4. Seasonal Variation and Molecular Subtype Interactions

Our two-way ANOVA analysis revealed a statistically significant interaction between molecular subtype and season of blood collection (F(3,81) = 3.70, *p* = 0.015), suggesting that seasonal variation in vitamin D status may differ across tumor subtypes. However, interpretation of this interaction is substantially complicated by an unexpected and problematic finding: the absence of a significant main effect of season on vitamin D concentrations (F(1,81) = 0.22, *p* = 0.64). This null finding for the main seasonal effect contradicts the extensive literature documenting pronounced seasonal fluctuations in 25(OH)D concentrations in temperate climate populations. Studies from similar latitudes demonstrate 20–40% variation between winter nadir and summer peak [12,22,23]. At Poland’s latitude (49–55° N), UVB radiation of sufficient intensity for cutaneous vitamin D synthesis is essentially absent from October through March, making seasonal variation biologically expected. The temporal relationship between vitamin D status and parathyroid hormone concentrations follows predictable seasonal trajectories in healthy populations, with vitamin D reaching the lowest levels in late winter when parathyroid hormone peaks [22].

Several factors may explain our unexpected absence of a main seasonal effect. First, when our already small subtype groups (*n* = 11–35) are further stratified by season, the resulting cell sizes (*n* = 3–18 per subtype–season combination) become extremely small, severely limiting statistical power to detect main effects or interactions reliably. Second, we did not systematically assess vitamin D supplementation use. If supplementation is more common during winter months, when individuals are aware of reduced sun exposure, this could obscure natural seasonal patterns. Third, the post-surgical timing of blood collection may have introduced temporal biases that disrupted typical seasonal patterns through surgery-related inflammatory responses, post-diagnosis behavioral changes, or treatment-related factors. Fourth, our convenience sampling across seasons may not have captured the full range of seasonal variation, particularly if sampling did not occur during the extreme nadirs or peaks of the annual cycle.

Given the absence of a demonstrable main effect of season consistent with the literature, the observed subtype × season interaction—despite reaching statistical significance—should be interpreted with considerable caution. It may represent a genuine biological phenomenon whereby tumor biology modulates seasonal vitamin D metabolism. Alternatively, and perhaps more likely given our study’s limitations, it could reflect a statistical artifact arising from multiple small-cell comparisons, unmeasured confounding by supplementation, temporal biases from post-surgical sampling, or chance findings in the context of limited power and multiple comparisons. Post-hoc pairwise comparisons did not reveal statistically significant differences between specific subtype–season combinations (all *p* > 0.05), though a trend was observed between HER2-enriched and luminal A subtypes during winter months (*p* = 0.067). The lack of statistically significant pairwise comparisons despite a significant interaction term further suggests that the interaction may be driven by complex patterns across multiple groups rather than discrete subtype-specific seasonal effects. Without a demonstrable main effect of season and with very small cell sizes in stratified analyses, we cannot confidently determine whether the observed interaction represents meaningful biology or methodological limitations. Future studies examining subtype-specific seasonal patterns should employ substantially larger samples with target cell sizes of *n* ≥ 30. These should include systematic documentation of vitamin D supplementation and sun exposure behaviors. Pre-diagnostic or at-diagnosis sampling would avoid post-treatment biases. Ideally, repeated measures across seasons within individuals would characterize temporal trajectories.

### 4.5. Correlates of Vitamin D Status

Age emerged as a statistically significant predictor of vitamin D insufficiency in multivariable logistic regression analysis, with each additional year associated with 6% increased odds (OR = 1.06, 95% CI: 1.01–1.12, *p* = 0.023). This age-related decline in vitamin D concentrations reflects well-established physiological changes, including reduced cutaneous synthesis capacity, decreased dietary intake, potential alterations in vitamin D metabolism, and reduced outdoor activity [19]. The modest magnitude of this association (6% per year) translates to approximately 60% higher odds of insufficiency for a 10-year age difference, which is clinically meaningful when considering population-level screening and supplementation strategies. BMI showed a borderline non-significant association (OR = 1.06, 95% CI: 0.99–1.14, *p* = 0.090), with each unit increase in BMI associated with 6% higher odds of vitamin D insufficiency. This relationship aligns with extensive literature documenting inverse associations between obesity and vitamin D status [24]. Proposed mechanisms include sequestration of fat-soluble vitamin D in adipose tissue, volumetric dilution in individuals with greater body mass, alterations in vitamin D metabolism, and potential behavioral factors such as reduced outdoor physical activity [25]. The borderline statistical significance in our analysis likely reflects limited sample size rather than the absence of association, as the effect size (OR = 1.06) is consistent with previous reports. Among breast cancer patients specifically, obesity represents a modifiable risk factor that may warrant consideration in vitamin D supplementation protocols, potentially requiring weight-adjusted dosing strategies.

Beyond vitamin D metabolism, obesity is associated with increased cancer risk across multiple sites through mechanisms involving adiposity-related inflammation, metabolic dysregulation, and hormonal alterations [26]. The co-occurrence of obesity and vitamin D insufficiency in breast cancer patients may represent converging pathways affecting cancer biology and prognosis. Dietary sources of vitamin D, including fatty fish, fortified dairy products, eggs, and meat, contribute to serum 25(OH)D concentrations alongside cutaneous synthesis [27]. Notably, meat products contain 25(OH)D directly rather than vitamin D3, providing bioavailable dietary sources particularly important during winter months when cutaneous synthesis is minimal [28]. Additionally, skeletal muscle appears to play a previously underappreciated role in storing and mobilizing vitamin D reserves during periods of limited UVB exposure [29]. Our study did not include a systematic dietary assessment, which represents an important limitation. Future investigations should comprehensively assess dietary vitamin D intake, supplementation use, and body composition to better characterize determinants of vitamin D status in breast cancer populations.

Season of blood collection did not significantly predict vitamin D insufficiency in our logistic regression model (OR = 1.20, 95% CI: 0.64–2.26, *p* = 0.58), consistent with the unexpected absence of a main seasonal effect observed in ANOVA analyses. As discussed above, this null finding contradicts biological expectations and published literature, likely reflecting study limitations rather than a genuine absence of seasonal variation in this population.

### 4.6. Clinical Implications

These findings have several implications for clinical practice, though they should be interpreted in light of study limitations. First, the widespread vitamin D insufficiency across both patient and control populations (55%) highlights the population-level public health challenge in Central European populations and suggests the potential need for systematic screening and supplementation protocols. Current evidence supports vitamin D supplementation for bone health in breast cancer patients, particularly those receiving aromatase inhibitor therapy, which further compromises bone mineral density [30]. Second, the null association between vitamin D status and molecular subtypes in our exploratory analysis—if confirmed in larger, adequately powered studies—would suggest that vitamin D screening and supplementation strategies need not vary based on tumor characteristics. However, given our borderline trend (*p* = 0.055) suggesting possibly higher vitamin D in HER2-enriched tumors, and emerging mechanistic evidence for subtype-specific VDR expression and signaling [15], this question remains open and warrants investigation in larger cohorts. Third, the associations with age and BMI indicate that these factors should inform vitamin D assessment and supplementation strategies. Older patients and those with elevated BMI may require more intensive screening and potentially weight-adjusted or age-adjusted dosing protocols, though optimal strategies require validation in clinical trials. Fourth, the high prevalence of insufficiency during winter months (even if not statistically different from summer in our limited sample) suggests that seasonal supplementation strategies warrant consideration, particularly in Northern European populations where UVB-mediated synthesis is absent for approximately half the year.

### 4.7. Study Limitations

Several important limitations warrant consideration in interpreting our findings. The most critical limitation is inadequate sample size, particularly for molecular subtype analyses. With only 11–35 patients per subtype, and further reduced to *n* = 3–18 per cell when stratified by season, our study lacks statistical power to reliably detect small to medium effect sizes. This severely underpowered design means that null findings should be interpreted as “insufficient evidence to detect associations” rather than “evidence of no association.” Adequately powered future studies would require 5–10 times our current sample size (approximately 80–100 patients per molecular subtype) to definitively characterize vitamin D status across subtypes.

Blood samples were obtained post-surgically. This introduces multiple biases. Surgical stress and inflammatory responses can acutely affect vitamin D metabolism. More fundamentally, post-diagnosis behavioral changes (reduced sun exposure due to fatigue or medical appointments, dietary modifications, supplement initiation, treatment-related factors) may have altered vitamin D status between cancer development and our assessment. If vitamin D plays a causal role in cancer development, our measurements do not reflect the etiologically relevant pre-diagnostic period. This temporal disconnect substantially limits causal inference and may explain null findings. Future studies should prioritize pre-diagnostic sampling from prospective cohorts or, at a minimum, sample collection at diagnosis prior to treatment initiation.

We did not systematically collect information on dietary vitamin D intake, supplementation use, or sun exposure behaviors. Dietary sources, including fatty fish, fortified dairy, and meat, contribute substantially to vitamin D status, particularly during winter months [27,28]. Vitamin D supplementation is increasingly common and could obscure associations with cancer or seasonal patterns. The absence of these data represents unmeasured confounding that limits interpretation. Future studies require a comprehensive assessment of all vitamin D sources.

The cross-sectional study design precludes causal inferences. We cannot determine whether observed vitamin D status preceded cancer development, resulted from cancer or treatment effects, or represents a coincidental association. Longitudinal designs with repeated measures before, during, and after cancer treatment are needed to characterize temporal trajectories and establish temporality.

Our unexpected finding of no significant main effect of season (*p* = 0.64), contradicting the extensive literature documenting 20–40% seasonal variation, raises concerns about the reliability of seasonal analyses in our data. This may reflect very small cell sizes when stratified (*n* = 3–18), unmeasured confounding by supplementation, post-surgical timing bias, or inadequate capture of seasonal extremes. The significant subtype × season interaction (*p* = 0.015) should therefore be interpreted with extreme caution and requires validation before biological interpretation.

We performed multiple statistical comparisons without formal adjustment for Type I error inflation (e.g., Bonferroni correction). This increases the probability of false-positive findings. However, applying corrections to our already severely underpowered analyses would further reduce power to detect true associations while appearing to enhance statistical rigor. We report unadjusted *p*-values with transparency about this limitation, consistent with the exploratory nature of this investigation. Future confirmatory studies should employ appropriate multiple testing procedures.

The study was conducted at a single institution in one geographic region (Lublin, Poland, latitude 51° N). Findings may not generalize to populations with different baseline vitamin D status, dietary patterns, genetic backgrounds, healthcare systems, or cancer screening practices. Multi-center studies across diverse populations are needed.

Genetic polymorphisms in genes involved in vitamin D metabolism (e.g., VDR, CYP2R1, CYP27B1, GC) can substantially influence serum 25(OH)D concentrations and may modify associations with cancer. We did not assess genetic factors, which represents unmeasured confounding.

### 4.8. Future Research Directions

Several research priorities emerge from this work.

Adequately powered studies with pre-diagnostic sampling: Large-scale prospective cohort studies with pre-diagnostic blood collection (or at minimum, at-diagnosis pre-treatment sampling) are essential. Target sample sizes of 80–100 patients per molecular subtype would provide 80% power to detect medium effect sizes (Cohen’s d = 0.5). Prospective designs would enable an assessment of whether pre-diagnostic vitamin D status influences cancer risk, molecular subtype distribution, or prognosis. Future studies should systematically document dietary vitamin D intake (with validated food frequency questionnaires), supplementation use (type, dose, compliance), sun exposure behaviors (time outdoors, sun protection practices, occupational exposure), genetic polymorphisms affecting vitamin D metabolism, and body composition (beyond simple BMI, including visceral adiposity assessment).

Repeated measures studies tracking vitamin D status before diagnosis (in prospective cohorts), at diagnosis, during treatment (surgery, chemotherapy, radiation, endocrine therapy), and through survivorship would characterize temporal dynamics and identify critical windows for intervention. This would address whether cancer or treatment alters vitamin D metabolism. Our borderline trend (*p* = 0.055), suggesting possibly higher vitamin D in HER2-enriched tumors, if validated, warrants mechanistic investigation. Studies should examine VDR expression levels, vitamin D signaling pathway activation, and functional consequences across molecular subtypes. This could inform personalized supplementation strategies.

Studies specifically designed to examine subtype-specific seasonal effects require much larger samples (target *n* ≥ 30 per subtype–season cell) and should include systematic supplementation assessment. Sampling should be strategically timed to capture seasonal extremes (February–March for nadir, August–September for peak).

Randomized controlled trials examining optimal vitamin D supplementation strategies for breast cancer patients should consider weight-adjusted dosing, treatment regimen-specific effects, and potential subtype-specific responses. Endpoints should include bone health, treatment tolerance, recurrence, and survival. Integration of vitamin D status with genomic, transcriptomic, and metabolomic profiling could identify vitamin D-responsive molecular signatures and subpopulations most likely to benefit from supplementation.

## 5. Conclusions

This exploratory cross-sectional study demonstrated widespread vitamin D insufficiency in Polish women regardless of breast cancer diagnosis, with 55% of participants maintaining serum 25(OH)D concentrations below 30 ng/mL. In analyses with important methodological limitations—including post-surgical blood sampling, small sample sizes (*n* = 11–35 per molecular subtype), and cross-sectional design—no statistically significant associations were observed between vitamin D status and breast cancer diagnosis or molecular subtype. A borderline non-significant trend (*p* = 0.055) suggested potentially higher 25(OH)D concentrations in HER2-enriched tumors compared to other subtypes, though this preliminary finding requires validation in adequately powered cohorts with pre-diagnostic or at-diagnosis sampling. Age emerged as a significant predictor of vitamin D insufficiency (OR = 1.06, *p* = 0.023), with BMI showing a borderline association (OR = 1.06, *p* = 0.090). The high prevalence of vitamin D insufficiency across both patient and control populations (55%) reflects the broader public health challenge in Central European populations and supports consideration of systematic screening and supplementation protocols, particularly for older individuals and those with elevated BMI.

However, whether vitamin D status influences breast cancer development, progression, or prognosis across molecular subtypes remains an open question requiring prospective studies with substantially larger sample sizes, pre-diagnostic blood collection, comprehensive assessment of dietary intake and supplementation use, and longitudinal follow-up. The primary contribution of this work lies in identifying critical methodological requirements for future research rather than providing definitive answers about vitamin D and breast cancer molecular subtypes.

## Figures and Tables

**Table 1:** Demographic and clinical characteristics of study participants.

Characteristic	Total (*n* = 168)	Breast Cancer (*n* = 89)	Controls (*n* = 79)	*p*-Value
Age (years), mean ± SD	54 ± 10	55 ± 10	53 ± 9	0.094
<50 years	56 (33)	28 (31)	28 (35)	
50–59 years	68 (40)	35 (39)	33 (42)	
≥60 years	44 (26)	26 (29)	18 (23)	
Menopausal status, *n* (%)				0.12
Premenopausal	57 (34)	27 (30)	30 (38)	
Postmenopausal	111 (66)	62 (70)	49 (62)	
BMI (kg/m^2^), mean ± SD	27 ± 5	27 ± 5	26 ± 5	0.50
Normal weight (18.5–24.9)	72 (43)	37 (42)	35 (44)	
Overweight (25.0–29.9)	63 (38)	33 (37)	30 (38)	
Obese (≥30.0)	32 (19)	18 (20)	14 (18)	
Season of collection, *n* (%)				0.10
Winter/Spring (Oct–Mar)	90 (54)	51 (57)	39 (49)	
Summer/Fall (Apr–Sep)	78 (46)	38 (43)	40 (51)	
Molecular subtype, *n* (%)				
Luminal A	—	35 (39)	—	
Luminal B	—	30 (34)	—	
HER2-enriched	—	11 (12)	—	
Triple-negative	—	13 (15)	—	

Data are presented as mean ± standard deviation (SD) or *n* (%). BMI, body mass index. *p*-values from independent *t*-tests (continuous variables) or chi-square tests (categorical variables).

**Table 2:** Serum 25(OH)D concentrations by group.

Group	*n*	Mean ± SD (ng/mL)	Median (ng/mL)	<30 ng/mL *n* (%)	*p*-Value
Breast cancer	89	30 ± 14	27.0	49 (55)	0.95 ^a^
Controls	79	29 ± 11	28.0	44 (56)	0.93 ^b^
Total	168	29.5 ± 12.8	28.0	93 (55)	

Data are presented as mean ± SD or *n* (%). 25(OH)D, 25-hydroxyvitamin D. ^a^ *p*-value from an independent *t*-test comparing the mean 25(OH)D between groups. ^b^ *p*-value from chi-square test comparing prevalence of insufficiency (<30 ng/mL).

**Table 3:** Vitamin D status by molecular subtype.

Molecular Subtype	*n*	Mean ± SE (ng/mL)	95% CI (ng/mL)	<30 ng/mL *n* (%)
Triple-negative	13	25.9 ± 3.8	18.5–33.4	7 (54)
HER2-enriched	11	37.6 ± 4.1	29.5–45.8	5 (45)
Luminal A	35	31.0 ± 2.3	26.5–35.5	18 (51)
Luminal B	30	26.4 ± 2.5	21.5–31.3	19 (63)
ANOVA:		F(3,81) = 2.64, *p* = 0.055		

Data are presented as mean ± standard error (SE) with 95% confidence intervals (CIs) or *n* (%). The borderline non-significant *p*-value (0.055) and wide confidence intervals indicate these findings should be interpreted cautiously, given the small sample sizes.

**Table 4:** Univariate ANOVA of vitamin D status by molecular subtype, season, and their interaction.

Effect	Univariate Tests of Significance for Vitamin D Status Sigma-Restricted Parameterization Effective Hypothesis Decomposition; Std. Error of Estimate: 12.9266				
	SS	Degr. of Freedom	MS	F	*p*
Intercept	60,360	1	60,360	360	<0.001
Molecular Subtype	1320	3	441	2.6	0.054
Season	37	1	37	0.22	0.64
Molecular subtype × season	1850	3	620	3.7	0.02
Error	13,500	81	167		

SS = sum of squares; MS = mean square; F = F-statistic; *p* = significance level.

**Table 5:** Logistic regression analysis of predictors of vitamin D deficiency in breast cancer patients.

Predictor	Level	OR	95% CI	*p*
Age (per year)	—	1.06	1.01–1.12	0.023
BMI (per kg/m^2^)	—	1.06	0.99–1.14	0.090
Season	Winter	1.20	0.64–2.26	0.58
Menopausal status	Postmenopausal	0.45	0.16–1.31	0.14
Group	Breast cancer	1.03	0.55–1.96	0.92

OR, odds ratio; CI, confidence interval; BMI, body mass index. Reference categories: Season = summer/fall; Menopausal status = premenopausal; Group = controls.

## Data Availability

Data supporting the conclusions of this article are available from the corresponding author upon reasonable request.
